# Exploring the ethnomycological potential of *Lentinus squarrosulus* Mont. through GC–MS and chemoinformatics tools

**DOI:** 10.1080/21501203.2019.1707724

**Published:** 2019-12-27

**Authors:** Reena Roy D, Shivananda Kandagalla, Krishnappa M

**Affiliations:** a Department of Applied Botany, Kuvempu University, Shivamogga, Karnataka, India; b Department of Biotechnology and Bioinformatics, Kuvempu University, Shivamogga, Karnataka, India; c Department of Computational Modeling of Drugs, Higher Medical and Biological School, South Ural State University, Chelyabinsk, Russia

**Keywords:** Wild mushrooms, *Lentinus squarrosulus*, mycocompounds, GC–MS, *in silico*, ADMET

## Abstract

Deciphering the ethnopharmacological importance is one of the prime steps towards understanding the indigenous traditional medicines practised over the centuries. With the advent of modern techniques, it is possible to unravel and explore the hidden ethnopharmacological benefits, comprising complex bioactive compounds of substantial health benefits and together it helps to treat the complex diseases without any side effects as seen in the case of modern synthetic drugs. In this concern, the present study aims to identify the ethnomycologically significant mycocompounds derived from the fruiting body of wild edible macrofungi, *Lentinus squarrosulus* that contain a vast array of compounds with notable edibility and a wide spectrum of medicinal applications. Proper authentication of mushroom taxonomy was exclusively done using macro and microscopic observations combining ITS DNA-based methods. Further, the isolate was subjected to fractionation in different solvent systems for mycochemical examination followed by GC–MS analysis. A total of 38 mycocompounds were identified through GC–MS and further subjected to *in silico* studies for drug-likeness, bioactivity and ADMET predictions to explore the druggability of mycocompounds. *In silico* analysis revealed 10 mycocompounds having good drug-likeness and ADMET properties. Altogether, the present study explored the ethnomycological potential of *L. squarrosulus* and identified potential mycocompounds.

## Introduction

The search for ethnopharmacologically important compounds from natural resources is gaining impetus attention in recent years in terms of beneficial health promotion with no side effects. Next to plants, the use of wild mushrooms has been used in folk medicines and as delicacy particularly for their taste and flavour for more than thousands of years. The wild mushrooms are natural reservoirs of most potent therapeutic mycocompounds because of their significant mycochemical compositions such as antioxidants, proteins, carbohydrates, lipids and numerous secondary metabolites that have attracted towards the development of mycopharmaceuticals.

Traditional knowledge of wild edible mushrooms is very important for the transfer of mycological knowledge to the new generations. Most of the ethnomycological applications are not easily accessible to the scientific community as the pieces of information are passed only through the word of mouth. Besides as food, the wild mushrooms are also used against various diseases especially the tribals and local people residing near to the forest vicinity. The bioactive potential of the mushrooms might have been attributed towards the presence of several pharmaceutically important mycocompounds that needs to be ascertained scientifically.

*Lentinus squarrosulus* is a palaeotropical species showing wide distribution extending throughout equatorial Africa, Southeast Asia, the Paciﬁc islands, and Australasia (Pegler 1983). It is a white-rot saprophytic fungus belonging to the family Polyporaceae. Morphologically, the basidiocarp is characterised by either whitish or greyish with notable conspicuous squamules on the surface (Njouonkou et al. 2013). The mushroom is usually found on fallen tree trunks, old stumps, and buried or exposed roots of trees. It usually grows in caespitose clusters, consisting of three to six basidiocarps, but sometimes, a tuft of up to 30 basidiocarps may be found (Mortimer et al. 2014).

The utility of *L. squarrosulus* remains underutilised despite the divergent uses reported from various parts of the country. It is therefore very essential to scientifically prove the medicinal uses that will certainly enhance the better understanding of bioprospecting of the encountered wild mushroom. The present study was therefore focused on the identification of ethnopharmacologically important mycocompounds from *L. squarrosulus*. In this regard, the isolated wild mushroom was subjected to GC–MS analysis followed by *in silico* analysis thereby extracting its important pharmaceutically potential mycocompounds of natural origin.

## Materials and methods

### Collection of macrofungi

**Figure 1.**
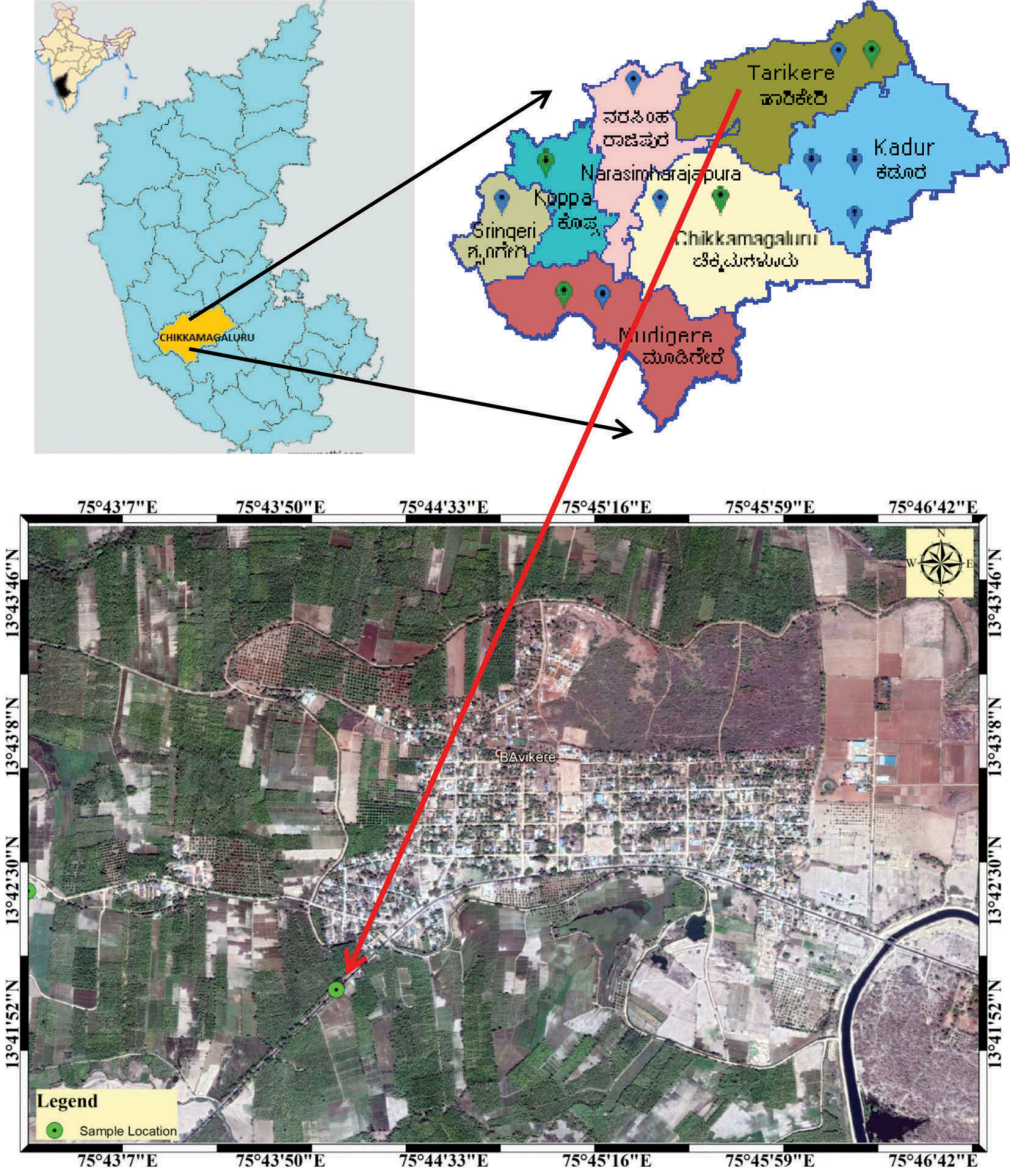
Geographical location map of *L. squarrosulus* collected at Bavikeretaluk, Chikkamagaluru district, Karnataka state, India.

An intensive field survey followed by mushroom collection was conducted during monsoon season near Bavikere taluk, Bhadra Wild Life Sanctuary of Chikkamagaluru district, a part of Central Western Ghats of Karnataka, India. The fruiting body of *L. squarrosulus* was collected from the tropical forest growing at 25ºC, 70% humidity and at an elevation of 662 m. Using Global Positioning System (GPS), the geographical coordinates (Latitude: 13°43’7.75“N and Longitude 75°42’32.62”E) were recorded and generated the maps (Figure 1).

### Authentication of *L. squarrosulus*

As reported in our previous work (Roy and Krishnappa 2018) the proper identity of wild species of *L. squarrosulus* was authenticated by morpho-molecular methods. The molecular identity of the macrofungi was ascertained based on the standard protocols. The nomenclature, taxonomic ranks and their associated descriptions and illustrations were accessed from the mycological databases like *Index Fungorum* (2017) and MycoBank (2017). The universal primers ITS1 and ITS4 (White et al. 1990) were used for the amplification of the ITS1-5.8S-ITS2 region. The 600–700 base pair ITS region of 5.8S rDNA was amplified using PCR polymerase. Sequence search was performed using the Basic Local Alignment Search Tool (BLAST) [https://blast.ncbi.nlm.nih.gov/genbank]. The newly obtained sequence with the identified specimen was then deposited at the NCBI GenBank and the accession number MH053154 was obtained (Roy and Krishnappa 2018).

### Nucleotide composition

The nucleotide base sequence percentage and histogram were analysed. The DNA sequences of macrofungi obtained were analysed using Sequencher software (version 5.2, Gene codes Corp, MD, USA) to generate the base frequency percentage and histogram. The four bases are Adenine (A), Thymine (T), Guanine (G) and Cytosine (C). The frequency report indicated the percentage of these bases.

### Preliminary physico-chemical and mineral elements (P, K, Ca, Mg, Fe, Mn, Zn and Cu) analysis

The powdered sample of *L. squarrosulus* was subjected to physico-chemical analysis. The foreign matter, moisture content, pH, water and alcohol soluble extractives, ash content, water-soluble ash, and acid-soluble ash were determined (Gupta 1984, 2003; Ahmad and Sharma 2001).

Further analysis of mineral elements in *L. squarrosulus* was evaluated. The element phosphorus (P) was estimated by vanado-molybdate yellow colour method and potassium (K) by flame photometer. Atomic absorption spectrophotometer (AAS) was used for the determination of calcium (Ca), magnesium (Mg), iron (Fe), manganese (Mn), zinc (Zn) and copper (Cu) in dried fruiting bodies of macrofungi (Bhargava and Raghupathi 1993). The values of elements P and K were expressed in terms of percentage and Ca, Mg, Fe, Mn, Zn and Cu were calculated as parts per million (ppm).

### Mycochemical extraction

The dried basidiocarp (fruiting body) of *L. squarrosulus* was subjected to Soxhlet method of extraction (Redfern et al. 2014) using petroleum ether, chloroform and ethanol depending on their dielectric constants from non-polar to polar system. The solvent was added to the round bottom flask which is attached to the soxhlet extractor and condenser. A constant temperature was maintained not exceeding 20ºC as excess of heating may lead to loss of thermolabile compounds. The process was allowed to run until colourless exudate was found from the extractor. The pooled extracts were then concentrated to dryness in vacuum using a rotary flash evaporator. The yield of the extracts was determined and stored in sterile screw-capped bottles in dark conditions at 4ºC. The extraction yield was expressed as,

$$\begin{array}{rl} & Extraction\;yield\left(\%\right)\\ & \;\;\;\;\;\;=\frac{Weight\;of\;the\;dry\;extracts\left(g\right)}{Weight\;of\;the\;sample\;used\;for\;extraction\left(g\right)}\times 100 \end{array}$$

### GC–MS analysis of mycocompounds

After extraction, the high yield fraction (Ethanol) was subjected to preliminary mycochemical screening to evaluate the composition of secondary metabolites and identified alkaloids, terpenoids, flavonoids, cardiac glycosides, steroids, phenols, proteins, fats and oils as the important chemical constituents which was reported in our previous work (Roy and Krishnappa 2018). Further, the fraction was subjected to Gas Chromatography–Mass Spectroscopy (GC–MS) analysis. The GC–MS (GC Model: Thermo Trace GC Ultra, MS Model: Thermo DSQ II, at Vittal Mallya Scientific Research Foundation, Bangalore, India) was done to identify the major bio-chemical constituents if any. The volatile non-iconic, thermally stable and low molecular weight compounds can be detected with the help of GC–MS. The volatile molecules are mainly responsible for the smell and taste of the mushrooms. The spectrum of the unknown components was compared with the spectrum of the known components in the mass spectral libraries at National Institute for Standards and Technology (NIST) and WILEY to identify the compounds. The chemical name, structure, molecular formula and molecular weight confirm the identification of the compounds. Peak areas and height indicate the concentration of various components present in the sample. The total GC–MS running time was 35 min.

### In silico analysis of mycocompounds

The structure information of identified mycocompounds derived from GC–MS analysis of ethanolic fraction of *L. squarrosulus* was mined using ChemSpider chemical database (http://www.chemspider.com/) and PubChem database (https://pubchem.ncbi.nlm.nih.gov/). From the chemical databases IUPAC (International Union of Pure and Applied Chemistry) name and structure information in different file format [SMILES (simplified molecular input line entry system)] were extracted for *in silico* analysis.

### Drug-likeness analysis and bioactivity prediction

The drug-likeness and bioactivity of identified mycocompounds were predicted using Molinspiration server (http://www.molinspiration.com). The drug-likeness properties such as molecular size, hydrophobicity, hydrogen bonding, electron distribution and other pharmacophore features influence directly on behaviour of compounds in terms of bioavailability, reactivity, toxicity and transportation. Together drug-likeness provides whether the compound of interest is similar to the known drug. The Molinspiration server calculates the molecular properties such as partition coefficient (milogP), topological polar surface area (TPSA), hydrogen bond donors (nOHNH) and acceptors (nOH), rotatable bonds (nrotb), number of atoms, molecular weight and volume on the basis of Lipinski’s rule of five (RO5) (Lipinski et al. 1997). Further, ADMET of mycocompounds were analysed using the web-based application, PreADMET (https://preadmet.bmdrc.kr/) as ADMET plays a significant role in the drug discovery process.

### In vitro antibacterial activity

The ethanolic fraction of *L. squarrosulus* was tested for antibacterial activity against three bacterial species, namely, *Staphylococcus aureus* (MTCC-902), *Escherichia coli* (MTCC-1599) and *Salmonella typhi* (MTCC-734) by agar well-diffusion method. All the bacterial cultures were maintained on nutrient agar (NA) and further subcultured in nutrient broth to reach the concentration of 10^6^ colony forming units (CFU)/ml. The cultures were incubated at 37ºC for 24 h.

The stock solution was prepared by dissolving ethanolic fraction in dimethyl sulphoxide (DMSO) at the maximum dissolved concentrations (100, 50, 25 mg/ml). Ciprofloxacin and DMSO served as positive and negative control, respectively. The NA was poured into each petri dish and bacterial cultures (100 μl) were inoculated aseptically. Wells of 5 mm diameter were punched in each plate using a sterile cork borer. After 20 min, the extract solution of 100 µl (25%, 50% and 100%) was added in respective wells. The antibacterial activity is measured in terms of diameter of zone of inhibition (mm).

### In vitro cytotoxicity analysis

The ethanolic fraction of *L. squarrosulus* was also tested for *in vitro* antiproliferative activity against the pancreatic cancer cell line using micro-titration colourimetric method of 3-(4,5-dimethylthiazol-2-yl)-2,5-diphenyltetrazolium bromide (MTT) cell viability assay (Mosmann 1983) with slight modifications. The MIA PaCa 2 cell line used in the present study was procured from National Centre for Cell Science, Pune. The cell line was grown in sterile Dulbecco’s Modified Eagle’s Medium (DMEM) and 10% Foetal Bovine Serum (FBS) at 37°C in a humidified 5% CO_2_ incubator. Briefly, MIA PaCa 2 cells were seeded into each of the 96 well plates at densities of approximately 1x10^4^cells/well at 37°C for 24 h. After 24 h of incubation, the cells were washed with fresh medium. The cells were then treated with extracts of different concentrations, 15.625, 31.25, 62.5, 125, 250, 500 and 1000 µg/ml and incubated for 24 h. Ten microlitres of MTT solution (5 mg/ml in phosphate buffer solution) was pipetted into each well and incubated for another 4 h. The medium was discarded and the formazan crystals were solubilised in DMSO for 30 min at 37°C in a CO_2_ incubator. Finally, using Elisa plate reader, the optical density (O.D) of the mixtures was measured at 570 nm. Triplicates were maintained for each sample concentration. The percentage of cell viability was calculated using the formula:

$$\begin{array}{rl} & Cell\;viability\;\%\\ & \;\;\;\;\;\;\;\;=\frac{Mean\;O.D\;of\;treated\;cells}{Mean\;O.\;D\;of\;untreated\;cells\;\left(control\right)}\times 100 \end{array}$$

A graph was plotted to enable the calculation of the inhibitory concentration (IC_50_) that kills the pancreatic cancer cells to that of control (untreated cells).

### Statistical analysis

The results obtained from the antibacterial activities of fruiting body extract of *L. squarrosulus* were subjected to statistical analysis. The experimental data were expressed as mean±SD with various concentrations against tested strains of bacteria. A one way ANOVA test and Tukey’s Multiple Comparison Test (p < 0.05) were used to determine the significant difference if any between the treatments.

## Results

### Nucleotide composition

**Figure 2.**
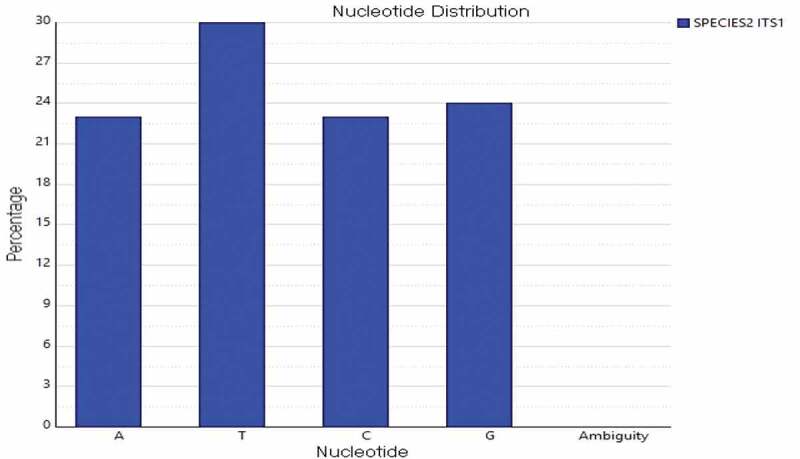
Percentage of nucleotide bases (ITS 1) of *L.squarrosulus.*

**Figure 3.**
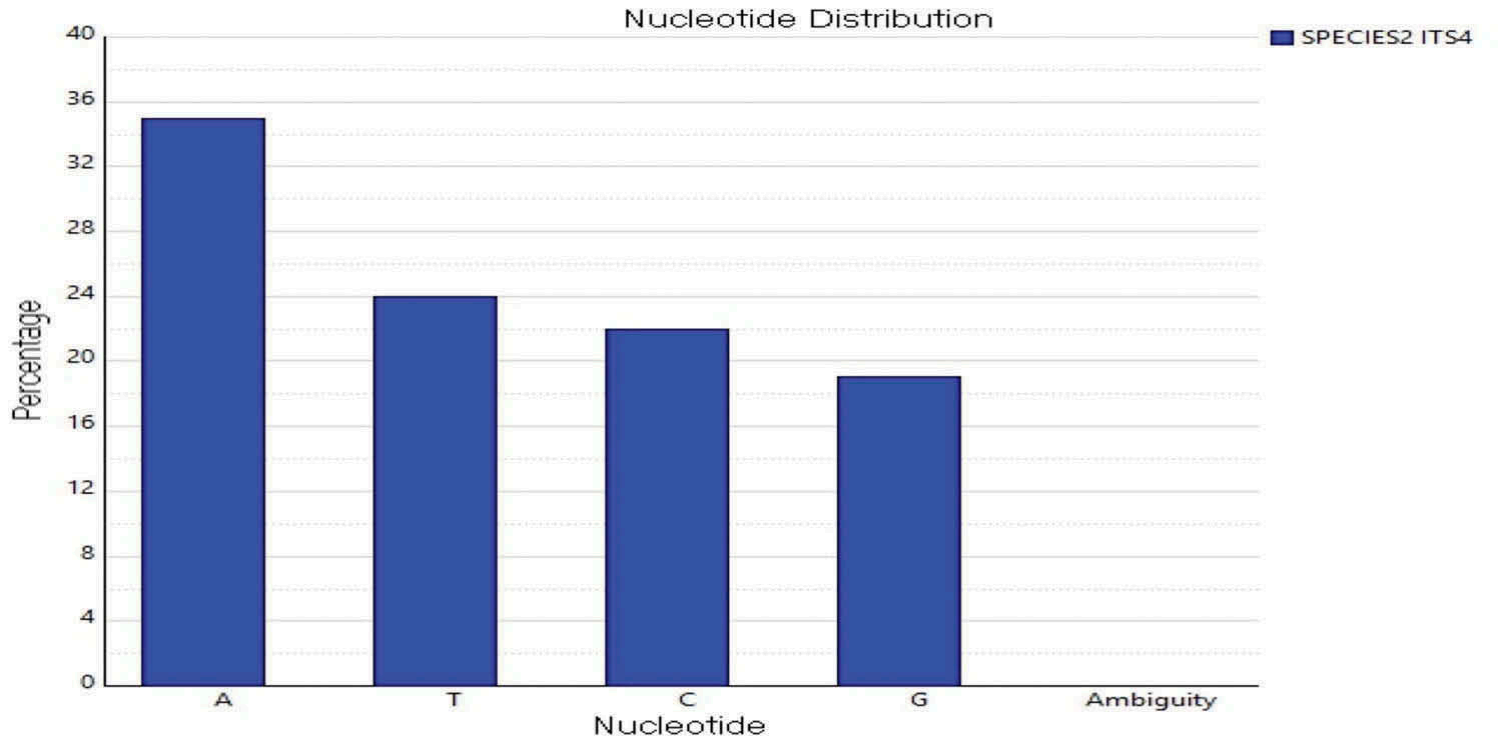
Percentage of nucleotide bases (ITS 4) of *L.squarrosulus.*

The DNA sequence of *L. squarrosulus* was analysed using Sequencher software and generated the base frequency percentage and histogram. The nucleotide base percentage of four bases Adenine (A), Thymine (T), Guanine (G) and Cytosine (C) for the amplification of ITS regions of *L. squarrosulus* using ITS1 and ITS4 primers was determined (Figures 2 and 3). The ITS region using ITS1 and ITS4 primers showed frequency percentage of A (23.11%), T (29.87%), C (23.43%), G (23.58%) and A (34.77%), T (24.08%), G (19.30%), C (21.85%), respectively. Together, the morphological and molecular identification using the ITS markers served as an effective tool. The molecular tools helped to assign taxonomic nomenclature to the wild mushroom. Furthermore, the ITS DNA methods solved the difficulties in identification posed in the ethnomycological studies.

### Preliminary physico-chemical and mineral elements analysis

The investigation on physico-chemical analysis showed that the foreign matter (should be less than 1.0%) and moisture content (should be less than 8.0% w/w) was found to be within the permissible limits in *L. squarrosulus*. The studied macrofungi showed a pH of 6. The water-soluble extractive values are more when compared to alcohol soluble indicating the presence of more polar soluble constituents such as acids, sugars and inorganic compounds. On dry weight basis, the total ash content was comparatively higher in *L. squarrosulus*. Collectively these results provided useful standards that aid in the genuine identity of the species (Complete details are shown in Table 1).

**Table 1.** Physico-chemical analysis of powdered basidiocarp of *L. squarrosulus.*

Sl.No	Parameters		*L. squarrosulus*
1.	Foreign matter (%w/w)		1.0
2.	Moisture content (%w/w)		0.01
3.	pH		6.7
4.	Extractive values (%w/w)	Water soluble extractive	31.2
		Alcohol soluble extractive	10.7
5.	Ash values (%w/w)	Total ash	25.12
		Water soluble ash	12.2
		Acid insoluble ash	67.2

Considering the mineral elements, the mushroom studied contained considerably high amount of minerals. The most abundant elements were K > P > Mg > Fe > Ca > Zn > Mn > Cu (Table 2). From these mineral elements analysis, it can be inferred that *L. squarrosulus* is an important source of essential nutrients.

**Table 2.** Essential elements in fruiting bodies of *L. squarrosulus.*

Minerals	*L. squarrosulus*
**Major elements (%)**	
Phosphorus	0.206
Potassium	0.726
Calcium	0.06
Magnesium	0.064
**Trace elements (mg/100 g)**	
Iron	35.47
Manganese	1.90
Zinc	2.25
Copper	1.5

### Mycochemical extraction

It was observed that the ethanol fraction produced maximum extractive yield (7.25%) followed by chloroform (0.68%) and petroleum ether (0.42%). Hence, ethanolic fraction was chosen for GC–MS analysis owing to high yield as well as due to the presence of more secondary metabolites as revealed from the preliminary mycochemical analysis.

### GC–MS analysis of mycocompounds

**Figure 4.**
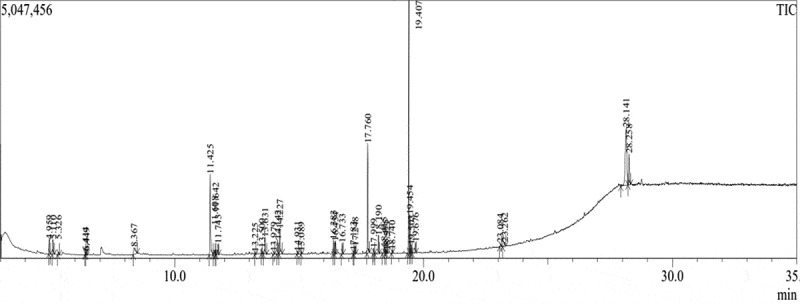
GC–MS chromatogram of ethanolic fraction of *L. squarrosulus.*

**Figure 5.**
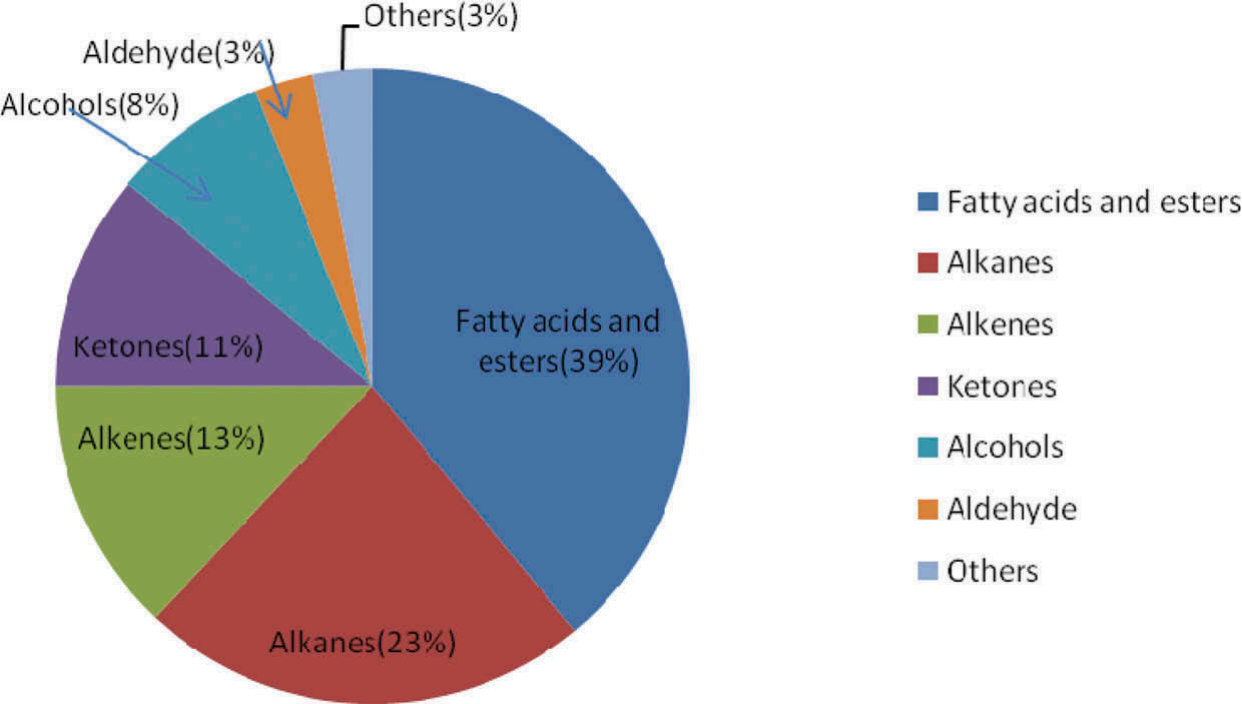
Percentage composition of the main groups of GC–MS analysed mycocompounds from ethanolic fraction of *L. squarrosulus.*

Analysis of GC–MS chromatogram of ethanolic fraction of *L. squarrosulus* showed 38 volatiles in varying proportions representing 38 different metabolites (Figure 4). All the mycochemicals with their compound name, retention time, molecular formula, molecular weight and concentration (peak area %) are presented in Table 3. The analysis recorded fatty acids and esters (39%), alkanes (23%), alkenes (13%), ketones (11%), alcohols (8%), aldehyde (3%) and others (3%), among which fatty acids and esters occupied the most prevailing mycocompounds and is represented in Figure 5. The most prevailing mycocompounds with high peak areas were fatty acid methyl esters like 9-Octadecenoic acid, methyl ester (4.18%), methyl 2-oxo-1-pyrrolidine acetate (10.40%), methyl palmitate (10.74%), methyl linoleate (24.21%) and a steroid ergosterol (16.98%). The complete mass spectral details of the most prevailing compounds were provided in the supplementary file (Supplementary file 1). The present study extracted mycocompounds in *L. squarrosulus* through GC–MS analysis and this eventually helps to infer the ethnopharmacologically important properties extracted traditionally.

**Table 3.** Compounds identified from the ethanolic fraction of *L. squarrosulus* through GC–MS.

Retention time (min)	Compound name	Mol. formula	Mol. weight	Peak area %
4.959	Hydroperoxide, 1-ethylbutyl	C_6_H_14_O_2_	118	1.39
5.110	Pentane, 3-ethyl-2, 4-dimethyl-	C_9_H_20_	128	1.47
5.326	3-Hexen-2-one	C_6_H_10_O	98	0.26
6.414	2(3H)-Furanone,dihydro-3-hydroxy-4,4-dimethyl-, (R)-	C_6_H_10_O_3_	130	0.41
6.449	2H-Pyran-2-one, 5,6-dihydro-	C_5_H_6_O_2_	98	0.30
8.367	2(3H)-Furanone, dihydro-4-hydroxy-	C_4_H_6_O_3_	102	1.90
11.425	Methyl 2-oxo-1-pyrrolidine acetate	C_7_H_11_NO_3_	157	10.40
11.608	2H-2,4a-Ethanonaphthalene, 1, 3, 4, 5, 6, 7-hexahydro-2, 5, 5-trimethyl-	C_15_H_24_	204	0.65
11.642	1-Dodecanol	C_12_H_26_O	186	0.49
11.743	Tetradecane	C_14_H_30_	198	1.06
13.225	(E,E,E)-3,7,11,15-Tetramethylhexadeca-1,3,6,10,14-pentaene	C_20_H_32_	272	0.21
13.500	2-Butenedioic acid (Z)-, dibutyl ester	C_12_H_20_O_4_	228	0.62
13.631	alpha-Caryophyllene	C_15_H_24_	204	2.02
13.979	Propylphosphonic acid, fluoroanhydride, 4-methylcyclohexyl ester	C_10_H_20_FO_2_P	222	0.29
14.142	1-Pentadecene	C_15_H_30_	210	1.34
14.227	n-Tetradecane	C_14_H_30_	198	3.42
14.931	n-Nonylcyclohexane	C_15_H_30_	210	0.26
15.089	8-Pentadecanone	C_15_H_30_O	226	0.24
16.383	1-Heptadecene	C_17_H_34_	238	1.37
16.454	Octadecane	C_18_H_38_	254	1.28
16.733	Pentadecanoic acid-methyl ester	C_16_H_32_O_2_	256	1.03
17.183	Cyclohexane, undecyl-	C_17_H_34_	238	0.28
17.248	1,2-Benzenedicarboxylic acid, dinonyl ester	C_26_H_42_O_4_	418	0.90
17.760	Hexadecanoic acid, methyl ester (Methyl palmitate)	C_17_H_34_O_2_	270	10.74
17.999	Benzenepropanoic acid, 3,5-bis(1,1-dimethylethyl)-4-hydroxy-, methyl ester	C_18_H_28_O_3_	292	0.52
18.190	Dibutyl phthalate	C_16_H_22_O_4_	278	2.36
18.415	1-Heptadecene	C_17_H_34_	238	0.80
18.475	n-Tricosane	C_23_H_48_	324	0.42
18.523	7-Hexadecenoic acid, methyl ester, (Z)-	C_17_H_32_O_2_	268	0.29
18.740	Hexadecanoic acid, 15-methyl-, methyl ester	C_18_H_36_O_2_	284	0.27
19.407	Methyl linoleate	C_19_H_34_O_2_	294	24.21
19.454	9-Octadecenoic acid, methyl ester	C_19_H_36_O_2_	296	4.18
19.503	9-Octadecenoic acid (Z)-, methyl ester	C_19_H_36_O_2_	296	0.92
19.676	Octadecanoic acid, methyl ester	C_19_H_38_O_2_	298	1.03
23.084	Hexadecanal	C_16_H_32_O	240	0.42
23.262	1,2-Benzenedicarboxylic acid, bis(2-ethylhexyl) ester	C_24_H_38_O_4_	390	0.63
28.141	Ergosterol	C_28_H_44_O	396	16.98
28.258	Ergosta-7,22-dien-3-ol, (3.beta.,5.alpha.,22E)-	C_28_H_46_O	398	4.62

### Analysis of mycocompunds derived from *L. squarrosulus*

The mycocompounds with IUPAC name of all the 38 mycocompounds (L1 – L38) procured from chemical database (ChemSpider) were detailed in the supplementary file-2.

### Drug-likeness analysis

Among 38 mycocompounds in *L. squarrosulus*, 10 mycocompounds, viz., **L1 (**Hydroperoxide, 1-ethylbutyl), **L3** (3-Hexen-2-one, 2), **L4** (2H-Pyran-2-one, 5,6-dihydro-), **L5 (**(3H)-Furanone, dihydro-3-hydroxy-4,4-dimethyl-), **L6** (2(3H)-Furanone, dihydro-4-hydroxy-), **L7** (Methyl 2-oxo-1-pyrrolidine acetate), **L8** (2H-2,4a-Ethanonaphthalene), **L12** (1,3,4,5,6,7-hexahydro-2,5,5-trimethyl-), **L14** (Propylphosphonic acid, fluoroanhydride, 4-methylcyclohexyl ester) and **L26** (Dibutyl phthalate) obeyed Lipinski’s rule of five. The complete results on drug – likeness analysis were furnished in the supplementary file-3. The mycocompounds which obeyed Lipinski’s rule will likely to be orally active and the miLogP (octanol-water partition coefficient) value of these mycocompounds was found to be within the acceptable range (< 5 Lipinski’s rule). The logarithm of the miLogP is very essential as it predicts the solubility of orally potential drug (Clark 1999).

TPSA (topological polar surface area) or PSA (polar surface area) is another molecular property that predicts the bioavailability of compounds. The prediction is based on surface sum of all polar atoms such as oxygen and nitrogen including hydrogen. It is a very good parameter indicating the drug distribution especially in illustrating human intestinal absorption (HIA), penetration to blood–brain barrier (BBB) and Caco-2 cell permeability (heterogeneous human epithelial colorectal adenocarcinoma cells). TPSA value greater than 140 Å has poor cell membrane permeability (Pajouhesh and Lenz 2005) and TPSA value of analysed compounds is in the range of 17.07–117.95 Å and is below the limit of 140 Å indicates good oral bioavailability. Further analysis of the number of hydrogen bond acceptors (nON) and the number of hydrogen bond donors (nOHNH) for all the mycocompounds were in accordance with the Lipinski’s ROF and ranged between 0 to 8 and 0 to 2, respectively, [range limit less than 10 and 5, respectively] (Lipinski 2004). The number of rotatable bonds of the analysed mycocompounds was within the range and some having more number of rotatable bonds. The number of rotatable bonds (≤ 10) indicates good bioavailability (Clark 1999). Together the analysis shows the druggable potential of mycocompounds isolated from *L. squarrosulus* of ethnomycological importance.

### Analysis of biological activity

Further bioactivity analysis of isolated mycocompounds exhibited very good to moderate activity based on its predicted bioactivity scores explored in terms of GPCR ligands, ion channel modulators, kinase inhibitors, nuclear receptor ligands, protease inhibitors and other enzyme targets from molinspiration server. Complete bioactivity scores of the analysed mycocompounds were tabulated in the supplementary file-4 and can be seen that if the bioactivity score is greater than 0.0, is most likely to exhibit considerable biological activities, score between −0.50 and 0.0 shows moderate activity and if the score is less than −0.50 then it is inactive state. The mycocompounds L14 (Propylphosphonic acid, fluoroanhydride, 4-methylcyclohexyl ester), L11 ((E,E,E)-3,7,11,15-Tetramethylhexadeca-1,3,6,10,14-pentaene), L13 (alpha.-Caryophyllene), L25 (Benzenepropanoic acid,3,5bis(1,1dimethylethyl)-4-hydroxy-), L31 (Methyl ester 9,12-Octadecadienoic acid (Z,Z)-), L35 (Methyl ester Hexadecanal), L37 (Ergosterol) and L38 (Ergosta-7,22-dien-3-ol, (3.beta.,5.alpha.,22E)) in *L. squarrosulus* showed very good to moderate biological activity.

### Analysis of ADMET properties

Further the mycocompounds were subjected to ADMET analysis to explore its potential at the phase of drug distribution. Those retaining weak ADME and higher toxicity properties get failed in the clinical phase. Also, this prediction reduces the risk of late-stage attrition and restricts in the screening of only the most promising mycocompounds. The server PreADMET predicts the HIA value (90–100%) and HIA plays a significant role during the initial stage of drug administration. Further server calculates PPB (Plasma Protein Binding) and BBB (Blood–Brain Barrier) of the compounds which play critical role in drug distribution phase. Also, server predicts the skin permeability, mutagenic and carcinogenic effect of the mycocompounds that determines the toxic effect. The rodent carcinogenicity studies are essential in identifying mycocompounds that are potentially hazardous to humans.

The ADMET properties of the mycocompounds with good drug-likeness scores are listed in Table 4. The L1, L3, L4, L5, L6, L7, L8, L12, L14 and L26 were well-absorbed compounds via intestinal tract as the HIA values for all the 10 mycocompounds are in the range of 80–100%. In addition, all these compounds showed average permeability to Caco2 cell and to MDCK cell model except L1. In PPB binding assessment, all the 10 mycocompounds exhibited weak binding with the plasma proteins (< 90%). The mycocompounds L3, L4, L5, L6, L7, L12, L14 and L26 showed low absorption to CNS (predicted value less than 0.1) and high absorption to CNS (predicted value above 2.0) for L1 and L8 in BBB penetration. The rate of skin permeability was least in all the compounds. All the mycocompounds showed mutagenic effect with positive results excluding L3, L4 and L6.

**Table 4.** Mycocompounds with good drug likeness and ADMET properties in *L.squarrosulus.*

								Toxicity		
	ADMET								Rodent carcinogenicity	
Name	Compound(ChemSpider ID)	HIA (%)	PPB (%)	BBB (%)	Caco2(nm/sec)	Skin permeability	MDCK (nm/s)	Ames test	Carcino_Mouse	Carcino_Rat
**L1(**Hydroperoxide, 1-ethylbutyl)	124448	93.92	96.12	2.06	13.32	−2.15	84.61	Mutagen	Positive	Negative
**L3(**3-Hexen-2-one)	4519228	100.0	86.45	0.79	42.900	−2.14	60.37	Mutagen	Negative	Negative
**L4(**2(3H)-Furanone, dihydro-3-hydroxy-4,4-dimethyl-)	964	86.58	94.46	0.40	20.49	−3.32	4.51	Mutagen	Negative	Negative
**L5(**2H-Pyran-2-one, 5,6-dihydro-)	454155	100	80.69	0.99	22.74	−2.94	51.23	Mutagen	Positive	Negative
**L6(**2(3H)-Furanone, dihydro-4-hydroxy-)	86345	83.49	78.88	0.35	15.39	−4.48	2.56	Mutagen	Negative	Negative
**L7(**Methyl 2-oxo-1-pyrrolidine acetate)	97873	91.39	28.37	0.43	21.17	−3.79	4.01	Mutagen	Positive	Positive
**L8(**2H-2,4a-Ethanonaphthalene, 1,3,4,5,6,7-hexahydro-2,5,5-trimethyl-)	521824	100.0	100.0	11.59	23.63	−1.31	43.96	Non-mutagen	Positive	Positive
**L12(**2-Butenedioic acid (Z)-, dibutyl ester)	4436356	95.53	100.0	0.43	26.64	−1.31	25.54	Mutagen	Positive	Positive
**L14(**Propylphosphonic acid, fluoroanhydride, 4-methylcyclohexyl ester)	505335	98.14	75.35	0.81	23.69	−1.29	43.59	Mutagen	Negative	Positive
**L26(**Dibutyl phthalate)	13837319	98.14	99.97	0.55	34.67	−1.69	3.30	Mutagen	Positive	Positive

### In vitro *antibacterial activity*

The ethanolic fraction of *L. squarrosulus* was analysed for antibacterial activity against three strains, namely, *E. coli, S. typhi* and *S. aureus* and the results showed promising activity against all the tested strains. The zone of inhibition against the bacterial species is listed in Table 5. Overall, our results showed the highest activity of *L. squarrosulus* at 100% concentration (2000 mg/ml) against *E. coli* (21.33 mm) followed by *S. typhi* (19.33 mm) and *S. aureus* (17.33 mm).

**Table 5.** Antibacterial activity of ethanolic fraction of *L. squarrosulus.*

Si.No.	Zone of Inhibition in mm (Mean±SD)					
	Bacterial strains	EA 25%	EA 50%	EA 100%	Standard Ciprofloxacin (1 mg/ml)	Control DMSO
1	*E. coli*	12.33 ± 0.17	15.1 ± 0.11	21.33 ± 0	18.07 ± 0	0 ± 0
2	*S. typhi*	14.27 ± 0	16.33 ± 0.11	19.33 ± 0.28	17.17 ± 0.28	0 ± 0
3	*S. aureus*	13.13 ± 0	15.17 ± 0	17.33 ± 0	18.33 ± 0.34	0 ± 0
One way ANOVA	P value	0.0001	0.0001	0.0001	0.0001	0.0001
	F value	154.5	154.5	154.5	154.5	154.5

Initial dilution of extract: 2000 mg/ml of DMSO (100%).

EA: Ethanolic extract; All the values were replicated three times; Statistical significance (P < 0.05).

### In vitro *cytotoxicity analysis*

**Figure 6.**
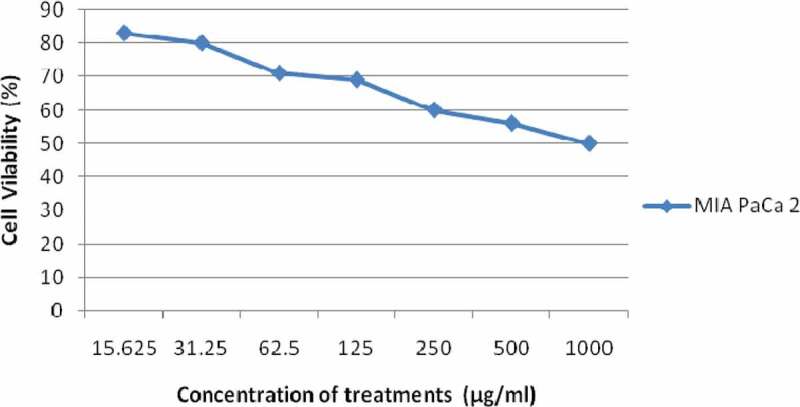
Cytotoxic effect of ethanolic fraction of *L. squarrosulus* against MIA PaCa 2 cancer cell lines at different concentrations (µg/ml) after 24 h of exposure.

The ethanolic fraction also displayed significant cytotoxicity against MIA PaCa 2 cell line. The present study evaluated the cytotoxicity as a measure of percentage cell death in a dose-dependent manner. Figure 6 illustrates the cytotoxic effect to 24-h exposure of pancreatic cancer cells to ethanolic fraction of *L. squarrosulus* to different concentrations. The cell viability was 83.49%, 79.53%, 71.23%, 69.20%, 60.42%, 55.98% and 50.48% at 15.625, 31.25, 62.5, 125, 250, 500 and 1000 µg/ml, respectively. With the increase in concentration of the extract the cancer cell number was reduced in MTT assay. It was observed that the extract exhibited significant reduction in cell viability in dose-dependent way against the selected cancer cell line when compared to that of untreated cells (Control) and the IC50 value was above 1000 µg/ml.

## Discussion

Extensive literature survey on ethnomycological potential of mushrooms showed great variations in their usages, depending on the people’s cultures and beliefs (Tibuhwa 2012; Harkonen et al. 2015). The use of *L. squarrosulus* either as food or medicine was utilised mainly by rural dwellers most notably the tribal communities. Dutta and Acharya (2014) reported that the local and tribal communities in West Bengal, India used *L. squarrosulus* mainly as food. The mushroom was used for treating mumps and heart diseases in Nigeria (Akpeja et al. 2003; Ayodele et al. 2009; Oyetayo 2011). It was also used by Nigerian folklore for treating anaemia and infertility in both men and woman (Okigbo and Nwatu 2015). The Bantu communities in Africa used this species in healing newborn baby’s navel (Van Dijk et al. 2003). The Irula communities in India utilised *L. squarrosulus* for treating fever, cough and fungal infections (Venkatacalapathi and Paulsamy 2016). Forest inhabitants of North West region of Cameroon used *L. squarrosulus* for system cleansing. All these indigenous medicinal uses clearly signify a plethora of mycocompounds with divergent medicinal uses. Hence, the research work was focused to collate the ethnomycological potential of *L. squarrosulus* in terms of the presence of active mycocompounds.

In the current study, the macrofungi identified showed strong connection and consistency to both morphology and molecular interpretation. The present study corroborates Avin et al. (2012) in which ITS sequences were used as an efficient taxonomic tool for identification of Basidiomycetes species. In the taxonomic point of view, the nucleotide composition proved to be immensely valuable as this information clarifies the proper authenticity of the target mushroom. Further physico-chemical analysis ensures the safety and confers guarantee for therapeutic quality (WHO (1998)). Altogether, from the physico-chemical analysis and the elemental analysis, it can be inferred that the basidiocarp of *L. squarrosulus* can provide as a useful source of essential nutrients thereby supporting the ethnomycological usage in the form of food reported so far.

In order to address the ethnomycological potential of *L. squarrosulus* in terms of the presence of active mycocompounds, we performed GC–MS analysis and identified a total of 38 mycocompounds. Further to explore the potential, these mycocompounds were subjected to *in silico* analysis and the study revealed that among 38 compounds, 10 mycocompounds showed good drug-likeness with ADMET properties. Additionally, we also performed antibacterial and cytotoxic activities of the ethanolic fraction of *L. squarrosulus* and results showed the potential of *L. squarrosulus* against different microorganisms and cancer cell lines.

Together, the present study attempted to understand the ethnomycological potential of *L. squarrosulus* in terms of the presence of active mycocompounds. Interestingly, our results clearly showed the presence of mycocompounds and among them very few compounds having good drug-likeness and ADMET properties. Apart from these mycocompounds we also observed the presence of different elements. Further analysis is required to evaluate the potential of these mycocompounds and also to test the synergistic effect of these mycocompounds with elements for ethnomycological activity.

## Conclusions

The search for ethnopharmacologically important compounds of fungal origin with possible bioactivities emphasising on important medicinal targets has been successfully accomplished in this study by multidisciplinary approaches employing modern technologies in the discovery of novel mycocompounds. The results demonstrated that *L. squarrosulus* possess remarkable pharmaceutical applications supporting the ethnomycological documentation of the experimented mushroom to treat various illnesses. The study evaluated 10 mycocompounds L1, L3, L4, L5, L6, L7, L8, L12, L14 and L26 with good drug-likeness and ADMET properties suggesting its hidden potential. Further, *in vitro* and *in vivo* studies are required to determine the mycochemical constituents of natural origin for validating its efficacy in treating the ethnomycologically documented ailments.

## References

[CIT0001] Ahmad RV, Sharma RK. 2001. Evaluation of drug for standardization. In: Proceedings of WHO, Pharmaceutical Lab for Indian Medicine. India: Ministry of Health and Family Welfare. Government of India, Ghaziabad.

[CIT0002] Akpeja EO, Isikhuemhen OS, Okhuoya JA. 2003. Ethnomycology and usage of edible and medicinal mushrooms among the Igbo people of Nigeria. Int J Med Mushrooms. 5(13):313–319.

[CIT0003] Avin FA, Bhassu S, Shin TY, Sabaratnam V. 2012. Molecular classification and phylogenetic relationships of selected edible Basidiomycetes species. Mol Bio Rep. 39:7355–7364. 22327649 10.1007/s11033-012-1567-2

[CIT0004] Ayodele SM, Akpaja EO, Adamu Y 2009. Some edible and medicinal mushrooms found in Igala land in Nigeria and their socio cultural and ethnomycological uses. In: Proceeding of the5th International Medicinal Mushroom Conference; Nantong, China. p. 526–531.

[CIT0005] Bhargava BS, Raghupathi HB. 1993. Analysis of plant materials for macro and micronutrients. In: Tandon HLS, editor. Methods of analysis of soils, plants, waters and fertilizers. New Delhi: FDCO; p. 49–82.

[CIT0006] Clark DE. 1999. Rapid calculation of polar molecular surface area and its application to the prediction of transport phenomena. J Pharm Sci. 88(8):815–821. 10430548 10.1021/js980402t

[CIT0007] Dutta AK, Acharya K. 2014. Traditional and ethno-medicinal knowledge of mushrooms in West Bengal, India. Asian J Pharm Clin Res. 7(4):36–41.

[CIT0008] Gupta AK. 1984. The ayurvedic system of medicine occurring in Charaka, Susrutha. Vol. II. New Delhi (India): Neeraj Publishing House.

[CIT0009] Gupta AK. 2003. Quality Standards of Indian Medicinal Plants. Vol. I. India: Indian Council of Medical Research.

[CIT0010] Harkonen M, Niemela T, Kotiranta H, Pierce G. 2015. Zambian mushrooms and mycology. Norrlinia. 29:1–208.

[CIT0011] Index Fungorum. 2017. [accessed 2017 8]. http://www.IndexFungorum.org.

[CIT0012] Lipinski CA. 2004. Lead- and Drug-like Compounds: the rule-of-five evolution. Drug Discov Today Technol. 1(4):337–341. 24981612 10.1016/j.ddtec.2004.11.007

[CIT0013] Lipinski CA, Lombardo F, Dominy BW, Feeney PJ. 1997. Experimental and computational approaches to estimate solubility and permeability in drug discovery and development settings. Adv Drug Delivery Rev. 46:3–26. 10.1016/s0169-409x(00)00129-0 11259830

[CIT0014] Mortimer PE, Xu J, Karunarathna SC, Hyde KD. 2014. Mushrooms for trees and people: A field guide to useful mushrooms of the Mekong region. World Agrofor East Asia Kunming China. 60:1–125.

[CIT0015] Mosmann T. 1983. Rapid colorimetric assay for cellular growth and survival: application to proliferation and cytotoxicity assays. J Immunol Methods. 65:55–63. 6606682 10.1016/0022-1759(83)90303-4

[CIT0016] MycoBank. 2017. http://www.MycoBank.org.

[CIT0017] Njouonkou AL, Mossebo DC, Akoa A. 2013. The genera *Lentinus* and *Panus* in the Dja biosphere reserve and its periphery, Cameroon. Kew Bulletin. 68:517–521.

[CIT0018] Okigbo RN, Nwatu CM. 2015. Ethnostudy and usage of edible and medicinal mushrooms in some parts of Anambra state, Nigeria. Nat Resour. 6:79–89.

[CIT0019] Oyetayo OV. 2011. Medicinal uses of mushrooms in Nigeria: towards full and sustainable exploitation. Afr J Tradit Complement Altern Med. 8(3):267–274. 22468005 10.4314/ajtcam.v8i3.65289 PMC3252220

[CIT0020] Pajouhesh H, Lenz GR. 2005. Medicinal chemical properties of successful central nervous system drugs. J Am Soc Exp Neurother. 2:541–553. 10.1602/neurorx.2.4.541 PMC1201314 16489364

[CIT0021] Pegler DN. 1983. The genus *Lentinus*: A world monograph, kew bulletin additional series. 10:1–281. London: H.M.S.O.

[CIT0022] Redfern J, Kinninmonth M, Burdass D, Verran J. 2014. Using Soxhlet ethanol extraction to produce and test plant material (essential oils) for their antimicrobial properties. J Microbiol Biol Educ. 15:45–46. 24839520 10.1128/jmbe.v15i1.656 PMC4004744

[CIT0023] Roy DR, Krishnappa M. 2018. Mycochemical profiling of *Lentinus squarrosulus* Mont., a wild edible macrofungi using GC-MS. Int J Pharm Sci Res. 9(10):4349–4354.

[CIT0024] Tibuhwa D. 2012. Folk taxonomy and use of mushrooms in communities around Ngorongoro and Serengeti National Park, Tanzania. J Ethnobiol Ethnomed. 8:36. 22999253 10.1186/1746-4269-8-36 PMC3539888

[CIT0025] Van Dijk H, Onguene NA, Kuyper TW. 2003. Knowledge and utilization of edible mushrooms by local populations of the rain forest of South Cameroon. AMBIO J Human Environ. 32:19–23. 12691487

[CIT0026] Venkatacalapathi A, Paulsamy S. 2016. Exploration of wild medicinal mushroom species in Walayar valley, the Southern Western Ghats of Coimbatore District Tamil Nadu. Mycosphere. 7(2):118–130.

[CIT0027] White TJ, Bruns T, Lee S, Taylor J. 1990. Amplification and direct sequencing of fungal ribosomal RNA genes for phylogenetics. In: Innis MA, Gelfand DH, Sninsky JJ, White TJ, editors. PCR protocols: a guide to methods and applications. New York (USA): Academic Press; p. 315–322.

[CIT0028] WHO. 1998. Quality control methods for medicinal plant materials. Geneva:World Health Organization Press.

