# Supplementary material for: Exploring the ethnomycological potential of Lentinus squarrosulus Mont. through GC–MS and chemoinformatics tools
Source: Mycology. 2019 Dec 27;11(1):78–89. doi: 10.1080/21501203.2019.1707724 (PMC7033700; doi:10.1080/21501203.2019.1707724)
Supplement: Supplemental Material [file TMYC_A_1707724_SM7630.zip › Supplemental files/Supplementary_file_1_.doc]

**Supplementary file 1:** Mass spectral details of all the 38 compounds identified from the ethanolic fraction of *Lentinus squarrosulus*

**Hydroperoxide, 1-ethylbutyl**

**Pentane, 3-ethyl-2, 4-dimethyl-**

**3-Hexen-2-one**

**2(3H)-Furanone,dihydro-3-hydroxy-4,4-dimethyl-, (R)-**

**2H-Pyran-2-one, 5,6-dihydro-**

**2(3H)-Furanone, dihydro-4-hydroxy-**

**Methyl 2-oxo-1-pyrrolidine acetate**

**2H-2,4a-Ethanonaphthalene, 1, 3, 4, 5, 6, 7-hexahydro-2, 5, 5-trimethyl-**

**1-Dodecanol**

**Tetradecane**

**(E,E,E)-3,7,11,15-Tetramethylhexadeca-1,3,6,10,14-pentaene**

**2-Butenedioic acid (Z)-, dibutyl ester**

**.alpha.-Caryophyllene**

**Propylphosphonic acid, fluoroanhydride, 4-methylcyclohexyl ester**

**1-Pentadecene**

**n-Tetradecane**

**n-Nonylcyclohexane**

**8-Pentadecanone**

**1-Heptadecene**

**Octadecane**

**Pentadecanoic acid-methyl ester**

**Cyclohexane, undecyl-**

**1,2-Benzenedicarboxylic acid, dinonyl ester**

**Hexadecanoic acid, methyl ester (Methyl palmitate)**

**Benzenepropanoic acid, 3,5-bis(1,1-dimethylethyl)-4-hydroxy-, methyl ester**

**Dibutyl phthalate**

**1-Heptadecene**

**n-Tricosane**

**7-Hexadecenoic acid, methyl ester, (Z)-**

**Hexadecanoic acid, 15-methyl-, methyl ester**

**Methyl linoleate**

**9-Octadecenoic acid, methyl ester**

**9-Octadecenoic acid (Z)-, methyl ester**

**Octadecanoic acid, methyl ester**

**Hexadecanal**

**1,2-Benzenedicarboxylic acid, bis(2-ethylhexyl) ester**

**Ergosterol**

**Ergosta-7,22-dien-3-ol, (3.beta.,5.alpha.,22E)-**
