# Supplementary material for: Exploring the ethnomycological potential of Lentinus squarrosulus Mont. through GC–MS and chemoinformatics tools
Source: Mycology. 2019 Dec 27;11(1):78–89. doi: 10.1080/21501203.2019.1707724 (PMC7033700; doi:10.1080/21501203.2019.1707724)
Supplement: Supplemental Material [file TMYC_A_1707724_SM7630.zip › Supplemental files/Supplementary_file_2.doc]

**Supplementary file 2:** Mycocompounds with IUPAC name and chemical identity number in *L. squarrosulus*

| **Compound name** | **IUPAC name** | **ChemSpider ID** |
| --- | --- | --- |
| **L1(**Hydroperoxide, 1-ethylbutyl) | 3-Hexanyl hydroperoxide | 124448 |
| **L2(**Pentane, 3-ethyl-2,4-dimethyl-) | 3-ethyl-2,4- dimethylpentane | 13421 |
| **L3(**3-Hexen-2-one) | (3E)-3-Hexen-2-one | 4519228 |
| **L4(**2(3H)-Furanone, dihydro-3-hydroxy-4,4-dimethyl-) | 3-Hydroxy-4,4- dimethyldihydro-2(3H)- furanone | 964 |
| **L5(**2H-Pyran-2-one, 5,6-dihydro- ) | 5, 6-Dihydro-2H-pyran-2-one | 454155 |
| **L6(**2(3H)-Furanone, dihydro-4-hydroxy-) | 4-hydroxyoxolan-2-one | 86345 |
| **L7(**Methyl 2-oxo-1-pyrrolidine acetate) | Methyl(2-oxo-1- pyrrolidinyl)acetate | 97873 |
| **L8(**2H-2,4a-Ethanonaphthalene, 1,3,4,5,6,7-hexahydro-2,5,5-trimethyl-) | 2,2,8- Trimethyltricyclo[6.2.2.0~1,6~]dodec-5-ene | 521824 |
| **L9(**1-Dodecanol) | 1-Dodecanol | 7901 |
| **L10(**Tetradecane) | Tetradecane | 11883 |
| **L11(**(E,E,E)-3,7,11,15-Tetramethylhexadeca-1,3,6,10,14-pentaene ) | (3E,6E,10E)-3,7,11,15- Tetramethyl-1,3,6,10,14-  hexadecapentaene | 4517825 |
| **L12(**2-Butenedioic acid (Z)-, dibutyl ester) | Dibutyl(2E)-2- butenedioate | 4436356 |
| **L13(**.alpha.-Caryophyllene) | (1E,4E,8E)-2,6,6,9-Tetramethyl-1,4,8-cycloundecatriene | 4444853 |
| **L14(**Propylphosphonic acid, fluoroanhydride, 4-methylcyclohexyl ester) | 4-Methylcyclohexyl propylphosphonofluoridate | 505335 |
| **L15(**1-Pentadecene) | 1-Pentadecene | 24142 |
| **L16(**n-Tetradecane) | Tetradecane | 11883 |
| **L17(**n-Nonylcyclohexane) | Nonylcyclohexane | 16908 |
| **L18(**8-Pentadecanone) | 8-Pentadecanone | 12609 |
| **L19(**1-Heptadecene) | 1-Heptadecene | 21723 |
| **L20(**Octadecane) | Octadecane | 11145 |
| **L21(**Pentadecanoic acid, methyl ester) | Methyl pentadecanoate | 21988 |
| **L22(**Cyclohexane, undecyl-) | Undecylcyclohexane | 37420 |
| **L23(**1,2-Benzenedicarboxylic acid, dinonyl ester) | Dinonyl phthalate | 6529 |
| **L24(**Hexadecanoic acid, methyl ester) | Methyl palmitate | 7889 |
| **L25(**Benzenepropanoic acid,3,5bis(1,1dimethylethyl)-4-hydroxy-, methyl ester) | Methyl3-4[hydroxyl-3,5- bis(2-methyl-2-propanyl) phenyl]propanoate | 56362 |
| **L26(**Dibutyl phthalate) | Dibutyl phthalate | 13837319 |
| **L27(**1-Heptadecene) | 1-Heptadecene | 21723 |
| **L28(**Tricosane) | Tricosane | 12017 |
| **L29(**7-Hexadecenoic acid, methyl ester, (Z)-) | Methyl(7Z)-7- hexadecenoate | 20121902 |
| **L30(**Hexadecanoic acid, 15-methyl-, methyl ester) | Methyl 15-methyl hexadecanoate | 455640 |
| **L31(**9,12-Octadecadienoic acid (Z,Z)-, methyl ester) | Methyl(9Z,12Z)-9,12- octadecadienoate | 4447491 |
| **L32(**9-Octadecenoic acid, methyl ester ) | Methyl 9-octadecenoate | 7910 |
| **L33(**9-Octadecenoic acid (Z)-, methyl ester**)** | Methyl(9Z)-9- octadecenoate | 4516661 |
| **L34(**Octadecanoic acid, methyl ester) | Methyl stearate | 7909 |
| **L35(**Hexadecanal ) | Palmitaldehyde | 959 |
| **L36(**1,2-Benzenedicarboxylic acid, bis(2-ethylhexyl) ester) | Bis(2-ethylhexyl) phthalate | 21106505 |
| **L37(**Ergosterol**)** | (3beta,22E)-Ergosta-5,7,22-trien-3-ol | 392539 |
| **L38(**:Ergosta-7,22-dien-3-ol, (3.beta.,5.alpha.,22E)) | (3beta,5alpha, 22E)- Ergosta-7,22-dien-3-ol | 4446722 |
