# Supplementary material for: Exploring the ethnomycological potential of Lentinus squarrosulus Mont. through GC–MS and chemoinformatics tools
Source: Mycology. 2019 Dec 27;11(1):78–89. doi: 10.1080/21501203.2019.1707724 (PMC7033700; doi:10.1080/21501203.2019.1707724)
Supplement: Supplemental Material [file TMYC_A_1707724_SM7630.zip › Supplemental files/Supplementary_file_4.doc]

**Supplementary file (Table 3):** Predicted biological activity scores of mycocompounds in *L. squarrosulus*

| **Compound No:** | **Compound (ChemSpider ID)** | **Biological activity** | | | | | |
| --- | --- | --- | --- | --- | --- | --- | --- |
|  |  | GPCR ligand | Ion channel modulator | Kinase inhibitor | Nuclear receptor ligand | Protease inhibitor | Enzyme inhibitor |
| **L1** | 124448 | -2.75 | -2.57 | -3.50 | -2.94 | -2.96 | -2.09 |
| **L2** | 13421 | -2.15 | -1.39 | -2.56 | -2.15 | -2.15 | -1.62 |
| **L3** | 4519228 | -3.56 | -3.36 | -3.83 | -3.34 | -3.45 | -3.01 |
| **L4** | 964 | -1.58 | -1.60 | -2.86 | -1.87 | -1.77 | -1.55 |
| **L5** | 454155 | -3.49 | -3.32 | -3.64 | -3.22 | -3.18 | -3.13 |
| **L6** | 86345 | -3.17 | -2.98 | -3.20 | -3.23 | -2.68 | -2.76 |
| **L7** | 97873 | -0.88 | -0.68 | -1.62 | -1.35 | -0.77 | -0.81 |
| **L8** | 521824 | -0.40 | -0.24 | -1.02 | -0.16 | -0.67 | 0.10 |
| **L9** | 7901 | -0.52 | -0.13 | -0.69 | -0.54 | -0.61 | -0.13 |
| **L10** | 11883 | -0.48 | -0.11 | -0.65 | -0.56 | -0.61 | -0.20 |
| **L11** | 4517825 | 0.02 | 0.15 | -0.20 | *0.41 | -0.21 | *0.47 |
| **L12** | 4436356 | -0.37 | -0.17 | -0.66 | -0.29 | -0.32 | -0.07 |
| **L13** | 4444853 | -0.14 | 0.02 | -0.93 | *0.34 | -0.67 | *0.31 |
| **L14** | 505335 | *0.41 | 0.10 | -0.68 | -0.62 | -0.03 | **1.04 |
| **L15** | 24142 | -0.38 | -0.06 | -0.66 | -0.32 | -0.48 | -0.09 |
| **L16** | 11883 | -0.48 | -0.11 | -0.65 | -0.56 | -0.61 | -0.20 |
| **L17** | 16908 | -0.31 | -0.05 | -0.59 | -0.39 | -0.37 | -0.09 |
| **L18** | 12609 | -0.35 | -0.10 | -0.73 | -0.22 | -0.36 | -0.01 |
| **L19** | 21723 | -0.21 | -0.01 | -0.45 | -0.14 | -0.30 | 0.00 |
| **L20** | 11145 | -0.14 | -0.00 | -0.26 | -0.17 | -0.23 | -0.00 |
| **L21** | 21988 | -0.17 | -0.06 | -0.42 | -0.16 | -0.20 | 0.01 |
| **L22** | 37420 | -0.15 | 0.00 | -0.39 | -0.20 | -0.20 | 0.00 |
| **L23** | 6529 | -0.03- | -0.06 | -0.13 | -0.00 | -0.05 | -0.03 |
| **L24** | 7889 | -0.11 | -0.05 | -0.34 | -0.09 | -0.13 | 0.04 |
| **L25** | 56362 | 0.07 | 0.09 | -0.22 | *0.35 | -0.04 | 0.15 |
| **L26** | 13837319 | -0.16 | -0.09 | -0.27 | -0.12 | -0.25 | -0.07 |
| **L27** | 21723 | -0.21 | -0.01 | -0.45 | -0.14 | -0.30 | 0.00 |
| **L28** | 12017 | 0.04 | 0.00 | -0.05 | 0.04 | 0.01 | 0.03 |
| **L29** | 20121902 | -0.05 | -0.03 | -0.36 | -0.03 | -0.13 | 0.11 |
| **L30** | 455640 | -0.06 | -0.03 | -0.33 | -0.02 | 0.03 | 0.04 |
| **L31** | 4447491 | 0.15 | 0.07 | -0.20 | 0.14 | 0.03 | *0.23 |
| **L32** | 7910 | 0.03 | -0.03 | -0.25 | 0.06 | -0.02 | 0.12 |
| **L33** | 4516661 | 0.03 | -0.03 | -0.25 | 0.06 | -0.02 | 0.12 |
| **L34** | 7909 | -0.03 | -0.04 | -0.23 | 0.00 | -0.03 | 0.05 |
| **L35** | 959 | -0.09 | *0.26 | -0.36 | -0.16 | -0.01 | 0.19 |
| **L36** | 21106505 | 0.01 | 0.01 | -0.13 | 0.07 | 0.04 | -0.01 |
| **L37** | 392539 | 0.14 | -0.13 | -0.34 | **0.74 | -0.08 | **0.53 |
| **L38** | 4446722 | 0.20 | 0.12 | -0.27 | **0.66 | 0.05 | **0.53 |
